# Assessing social needs among patients with cardiovascular and psychiatric comorbidities in free community health clinics

**DOI:** 10.1371/journal.pone.0291682

**Published:** 2023-09-19

**Authors:** David Haddad, Venkata Sai Jasty, Jacob Ref, Paul Hsu, Patricia Lebensohn, Tze-Woei Tan

**Affiliations:** 1 Department of Orthopaedic Surgery, The University of Arizona College of Medicine-Tucson, Tucson, Arizona, United States of America; 2 Department of Internal Medicine, The University of Michigan Medical School, Ann Arbor, Michigan, United States of America; 3 The University of Arizona College of Medicine-Tucson, Tucson, Arizona, United States of America; 4 The University of Arizona, College of Public Health, Tucson, Arizona, United States of America; 5 Department of Family and Community Medicine, The University of Arizona College of Medicine-Tucson, Tucson, Arizona, United States of America; 6 Division of Vascular Surgery, Keck School of Medicine of University of Southern California, Los Angeles, California, United States of America; The University of Mississippi Medical Center, UNITED STATES

## Abstract

**Background:**

Community-related health assessments have been shown to improve several outcomes in socioeconomically disadvantaged populations with comorbid chronic health conditions. However, while it is recognized that modifiable social determinant of health (SDH) factors might be responsible for up to 60% of preventable deaths, it is not yet standard of care to routinely screen and address these at preventive health appointments. The objective of this study was to identify the social needs of socioeconomically disadvantaged patients.

**Methods:**

We performed a retrospective review of the socioeconomic screening questionnaires distributed to under- and uninsured patients seen at a medical student-run free primary care-based community clinic. This study included participants of all ages (0 and up), genders, languages, and ethnicities who filled out the social screening questionnaire. Socioeconomic screening questionnaires assessed the need for critical resources such as food, housing, utilities, finances, transportation, childcare, employment, education, legal support, companionship, health literacy, and community assistance. The primary study outcome was to identify unmet social needs of our medical student-run free clinic patients. We secondarily sought to identify associations between these needs and chronic health conditions. We hypothesized that patients with multiple chronic health problems and financial stressors would have the highest requests for resources.

**Results:**

Our retrospective review identified 264 uninsured participants who were evaluated for social needs using a screening questionnaire. Participants who reported unmet social needs had significantly more cardiovascular risk factors than those who did not. Cardiovascular comorbidities and a history of psychiatric illness were the two most common medical problems significantly associated with several unmet social needs.

**Conclusion:**

This study provides support for the preemptive identification and appropriate management of physical, mental, and social care to improve disproportionate disparities in long-term health outcomes.

## Introduction

Access to health insurance is critical to receiving preventive care and an improved quality of life [1]. The number of uninsured people in the United States (U.S.) has historically been high but rose to more than 30 million in 2020 as the COVID-19 pandemic caused an economic recession and massive unemployment rates [2]. This global crisis has consequently amplified the health inequities and poor outcomes already burdening disadvantaged patient populations with chronic cardiovascular and psychiatric diseases [3, 4]. To meet the rising rates of these illnesses, medical student-run free clinics (SRFC) have become an increasingly more prevalent method of providing primary care services to low-income and medically underserved patients across the U.S. [5] In addition to providing medical care, these free clinics further offer a unique opportunity to address the underlying social risk factors that perpetuate long-term, inequitable health outcomes [6].

The World Health Organization describes the social determinants of health (SDH) as the conditions in which people are born, grow, work, live, and age [7]. However, while it is recognized that modifiable SDH factors are responsible for up to 60% of preventable deaths, it is not yet standard of care to routinely screen and address these at preventive health appointments [6, 8–10]. In 2019, our team implemented a social intervention program to better identify and manage the adverse SDH factors providing disproportionally high barriers to care for patients seen at our SRFCs. This innovative organization, called Care Connections, mobilizes medical students as social workers to routinely screen and assist patients using social need questionnaires based on literature proven social intervention programs [11, 12]. Our method specifically capitalizes on the developing clinical acumen of medical students to alleviate overburdened caregivers and the healthcare system, and also holistically manage individual social risks that worsen chronic health conditions [4, 13].

Integration of these services into SRFCs is critical given the increasing volume of patients precipitated by financial and medical limitations imposed by the COVID-19 pandemic. In addition, our model facilitates early real-world clinical and community outreach opportunities for medical students to connect with patients at the highest risk of COVID related complications [14]. Our objective was to perform a retrospective review to identify the social needs of our SRFC patients and improve the administration of community health referrals within our clinics. We hypothesized that patients with more chronic health problems and financial stressors would have the highest requests for resources. Community related health interventions have been shown to improve several outcomes in underinsured populations with comorbid chronic health conditions [1]. However, it is crucial to better understand how routine SDH screening tools can be implemented to efficiently guide more individualized, comprehensive care to the marginalized and vulnerable patient populations seen in free community health clinics.

## Methods

### Ethics statement

This study was approved by the University of Arizona Institutional Review Board (Study reference: 00000648) and a Health Insurance Portability and Accountability Act waiver of informed consent was granted due to our de-identification of reported data.

### Setting

We performed a retrospective review of the socioeconomic screening questionnaires distributed at the University of Arizona College of Medicine–Tucson’s (UACOM-T) SRFCs from August 1^st^, 2019 to April 1^st^, 2022. Our institution serves patients with limited or no access to health care through the medical student-run and directed Commitment to Underserved People (CUP) Program [15, 16]. First implemented as an elective seminar in 1978 [15], this umbrella initiative has grown to encompass several primary care based clinics including those devoted to mental health, refugees, LGBT+ patients, integrative medicine, family care, pediatrics, and women’s health [16]. These privately funded SRFCs are directed by first-year medical students under the supervision of faculty, typically operate on weekday evenings or weekend mornings, and can either be performed in person at the medical campus or via telehealth. Located near the Mexico-Arizona border, our clinics serve a large, diverse population of medically underserved patients.

In the standard workflow, patients are greeted by clinic intake personnel who gather demographic information and distributes the social screening form (Table 1). The patient is then delegated to a clinical care team, which consists of a preclinical (first or second-year) medical student paired with a clinical (third or fourth-year) medical student. Prior to clinical evaluation, the patients who opted to fill out the social screening tool are interviewed by the first-year representative from our social intervention group, Care Connections. Interview and SDH screening questions (Table 1) are based on the open-source models published by Health Leads [12] and the Social Intervention Research and Evaluation Network’s Gravity Project [11]. Care Connections then debrief with the care team after their medical evaluation and presentation to the attending physician. A comprehensive list of community resources is subsequently assembled for the patient according to their unmet needs. Notes and screening tool results are transcribed into the free cloud-based Electronic Health Record (EHR) platform Practice Fusion (Version 3.7.1.192.0.4608, Practice Fusion, Inc, San Francisco, CA).

**Table 1:** Socioeconomic screening questions employed at clinics.

Food insecurity: In the last 12 months, did you ever eat less than you felt you should because there wasn’t enough money for food?
Housing insecurity: Are you worried or concerned that in the next two months you may not have stable housing that you own, rent, or stay in as part of a household?
Utility need: In the past 12 months has the electric, gas, oil, or water company threatened to shut off services in your home?
Financial resources: In the last 12 months, was there a time when you needed to see a doctor but could not because of cost?
Transportation insecurity: In the last 12 months, have you ever had to go without health care because you didn’t have a way to get there?
Childcare: Do problems getting childcare make it difficult for you to work or study?
Employment instability: During the last four weeks, have you been actively looking for work or a job training program?
Education: Are you currently looking to go back to school to earn a higher education? (eg. GED, CNA/CMA, etc.)
Legal resources: Do you currently need help acquiring legal services? (ex. domestic abuse, housing, etc.)
Social isolation: Do you often feel that you lack companionship?
Education: Do you ever need help reading hospital materials?
If checked yes to any boxes above, would you like to receive assistance with any of these needs?

### Participants

We included patients of all ages (0 and up), genders, languages, and ethnicities who were treated at the previously discussed SRFCs and who filled out the social screening questionnaire. Children (under the age of 18) were required to be accompanied by a legal guardian or adult appointed to provide consent for evaluation. Patients who spoke a language other than English were interviewed with the assistance of a certified interpreter. We excluded patients who filled out a questionnaire but were not seen immediately after by a medical team at the same visit (n = 9).

### Variables

Patient charts were reviewed to collect the following variables: Age, sex, language (Spanish, English, or neither), ethnicity (Hispanic/Latino or non-Hispanic/Latino), number and type of comorbidities, number and type of medications, active tobacco use, active alcohol use, active illicit drug use, and number of follow up visits. All diagnostic criteria were determined at the time of appointment by the treating physicians and subsequently recorded in Practice Fusion. The study sample size was determined by the number of patients who filled out a social screening form and the information available in the chart. Collected data was anonymized and de-identified prior to analysis. The primary outcomes were identifying community members and their social needs within our free clinic, then understanding the association of these variables with contributing risk factors and chronic diseases. Secondary outcomes consisted of analyzing the labs and vitals of these patients in relation to the unmet social needs documented during their subsequent clinic visit.

### Statistical methods

Social needs were assessed in relation to patient characteristics and comorbidities/medications using univariate analyses. All continuous variables except for the number of follow ups, number of comorbidities, and number of medications were summarized by mean and analyzed with Wilcoxon rank-sum tests to compare between groups. These three exceptions were instead summarized by median (interquartile range) and Wilcoxon rank-sum tests were performed to compare each of them between groups. Binary variables were summarized by frequency and Fisher’s exact tests were performed to compare each of them between groups. Statistical significance for all analyses was set at a p-value of <0.05.

## Results

Our query identified 273 total patients who were evaluated for social needs using our screening questionnaire. Nine of these patients were excluded due to not meeting our inclusion criteria. The mean age of patients was 39.9 (0.4–90), and there were 177 females (67.0%) and 87 males. All participants were uninsured. Eighty-eight patients (33.3%) identified themselves as Hispanic/Latino and 176 patients identified as Non-Hispanic/Latino (Table 2). Of the included 264 patients, 63% requested a community referral to receive resources for financial support, which was also the most frequently reported unmet social need (62%). The least requested unmet need was related to legal resources (5.7%) (Table 3).

**Table 2:** Characteristics patients who filled out the socioeconomic screening questionnaire.

Patient Characteristics	N = 264	%
Mean Age	39.9 ± 1.01 (28.00, 53.00)	-
Median follow-ups	1.75 ± 0.17 (0.00, 2.00)	-
Median Number of medications	1.90 ± 0.14 (0.00, 3.00)	-
Median Number of comorbidities	3.43 ± 0.14 (2.00, 4.00)	-
**Sex**		
Female	177 ± 0.03	67.05%
Male	87 ± 0.03	32.95%
**Ethnicity**		
Non-Hispanic/Latino	176 ± 0.04	66.67%
Hispanic/Latino	88 ± 0.04	33.33%
**Language**		
English	136 ± 0.03	51.52%
Spanish	72 ± 0.05	27.27%
Other	56 ± 0.03	21.21%
**Types of Comorbidities**		
Psychiatric	88 ± 0.04	33.33%
Alcohol	87 ± 0.04	32.95%
Tobacco	72 ± 0.04	27.27%
Hypertension	58 ± 0.04	21.97%
Diabetes Mellitus	57 ± 0.05	21.59%
Dyslipidemia	45 ± 0.05	17.05%
Illicit Drugs	43 ± 0.04	16.29%
**Active at Initial Encounter**		
Hypertension Medication	45 ± 0.05	77.59%
Psychiatric Medication	43 ± 0.06	48.86%
Diabetes Mellitus Medication	31 ± 0.07	54.39%
Dyslipidemia Medication	14 ± 0.00	31.11%
Aspirin	11 ± 0.00	19.30%

**Table 3:** Summary of responses from patients who filled out the socioeconomic screening questionnaire.

Reported Unmet Needs	N	%
Total	264 ± 0.01	-
Requested resources	166 ± 0.03	63.00%
Finances	163 ± 0.04	62.00%
Employment	57 ± 0.04	22.00%
Food	55 ± 0.04	21.00%
Education	53 ± 0.04	20.00%
Transportation	43 ± 0.04	16.00%
Utility services	41 ± 0.04	16.00%
Companionship	39 ± 0.04	15.00%
Housing	37 ± 0.04	14.00%
Health literacy	31 ± 0.04	12.00%
Childcare assistance	16 ± 0.03	6.10%
Legal	15 ± 0.03	5.70%

Univariate analyses (Tables 4 and 5) revealed that more patients with a history of illicit drug use (27.27% vs 13.40%, p = 0.022), musculoskeletal (MSK) conditions (61.82% vs 42.11%, p = 0.010), psychiatric illness (63.64% vs 26.32%, p<0.001), psychiatric medications (30.91% vs 12.44%, p = 0.002), or a higher mean number of comorbidities (4 vs 3, p<0.001) had a significant association with food insecurity compared those without. Patients were significantly more likely to report unmet housing needs if they had a history of alcohol use (48.65% vs 30.40%, p = 0.037), tobacco use (45.95% vs 24.23%, p = 0.009), speaking English (72.97% vs 48.02%, p = 0.007), psychiatric illness (62.16% vs 29.52%, p<0.001), or a higher mean number of comorbidities (5 vs 3, p<0.001). A history of psychiatric illness (58.54% vs 29.60%, p<0.001), or a higher mean number of comorbidities (4 vs 3, p = 0.010) were significantly associated with unmet utility needs. Patients who reported needing financial resources were significantly associated with illicit drug use (21.47% vs 7.92%, p = 0.003) and a higher mean number of comorbidities (>3 vs <3, p = 0.028).

**Table 4:** Summary of patient characteristics by various unmet social needs.

Patient Characteristics		Female	Hispanic/Latino	Spanish	English	Illicit drug use	Tobacco use	Alcohol use	Age
Financial	N = 163	104 (63.80%)	53 (32.52%)	42 (25.77%)	89 (54.60%)	35 (21.47%)	47 (28.83%)	60 (36.81%)	39.00 (28.50, 54.00)
	p-value	0.18	0.79	0.57	0.21	0.003	0.48	0.11	0.24
Employment	N = 57	41 (71.93%)	16 (28.07%)	12 (21.05%)	30 (52.63%)	12 (21.05%)	19 (33.33%)	27 (47.37%)	34.00 (28.00, 50.00)
	p-value	0.43	0.43	0.31	0.88	0.31	0.25	0.011	0.2
Food security	N = 55	34 (61.82%)	16 (29.09%)	12 (21.82%)	31 (56.36%)	15 (27.27%)	18 (32.73%)	19 (34.55%)	37.00 (26.50, 50.50)
	p-value	0.42	0.52	0.4	0.45	0.022	0.31	0.87	0.41
Education	N = 53	40 (75.47%)	17 (32.08%)	14 (26.42%)	33 (62.26%)	13 (24.53%)	16 (30.19%)	23 (43.40%)	30.00 (26.00, 46.00)
	p-value	0.19	0.87	>0.99	0.092	0.094	0.61	0.075	0.012
Transportation	N = 43	29 (67.44%)	12 (27.91%)	9 (20.93%)	29 (67.44%)	11 (25.58%)	13 (30.23%)	19 (44.19%)	35.00 (26.00, 49.50)
	p-value	>0.99	0.48	0.35	0.03	0.11	0.71	0.11	0.14
Utilities	N = 41	28 (68.29%)	14 (34.15%)	13 (31.71%)	24 (58.54%)	6 (14.63%)	14 (34.15%)	18 (43.90%)	41.00 (26.00, 50.00)
	p-value	>0.99	>0.99	0.57	0.4	>0.99	0.34	0.11	0.79
Companionship	N = 39	25 (64.10%)	14 (35.90%)	13 (33.33%)	19 (48.72%)	7 (17.95%)	10 (25.64%)	15 (38.46%)	41.00 (30.50, 54.00)
	p-value	0.71	0.72	0.44	0.73	0.81	>0.99	0.46	0.37
Housing	N = 37	22 (59.46%)	10 (27.03%)	8 (21.62%)	27 (72.97%)	10 (27.03%)	17 (45.95%)	18 (48.65%)	41.00 (27.00, 50.00)
	p-value	0.35	0.45	0.55	0.007	0.089	0.009	0.037	0.83
Health literacy	N = 31	26 (83.87%)	17 (54.84%)	14 (45.16%)	14 (45.16%)	5 (16.13%)	9 (29.03%)	10 (32.26%)	47.00 (34.50, 62.50)
	p-value	0.041	0.014	0.03	0.57	>0.99	0.83	>0.99	0.012
Childcare assistance	N = 16	9 (56.25%)	5 (31.25%)	4 (25.00%)	9 (56.25%)	4 (25.00%)	5 (31.25%)	5 (31.25%)	32.00 (27.50, 35.75)
	p-value	0.41	>0.99	>0.99	0.8	0.31	0.77	>0.99	0.022
Legal	N = 15	11 (73.33%)	6 (40.00%)	6 (40.00%)	10 (66.67%)	2 (13.33%)	3 (20.00%)	7 (46.67%)	33.00 (29.00, 41.50)
	p-value	0.78	0.58	0.25	0.29	>0.99	0.77	0.27	0.3
Received help	N = 166	115 (69.28%)	58 (34.94%)	49 (29.52%)	86 (51.81%)	24 (14.46%)	46 (27.71%)	57 (34.34%)	38.00 (28.00, 52.75)
	p-value	0.34	0.5	0.32	>0.99	0.31	0.89	0.59	0.84

**Table 5:** Summary of patient comorbidities and medication uses by various unmet social needs.

Comorbidities and Medications		Gastrointestinal Disease	Musculoskeletal Conditions	Psychiatric Illness	Diabetes Mellitus	Hypertension	Dyslipidemia	Taking Aspirin	Taking Hypertension Medication	Taking Diabetes Mellitus Medication	Taking Psychiatric Medication	Taking Dyslipidemia Medication	Number of Follow Ups	Number of Comorbidities	Number of Medications
**Financial**	**N = 163**	56 (34.36%)	82 (50.31%)	56 (34.36%)	37 (22.70%)	33 (20.25%)	28 (17.18%)	5 (3.07%)	27 (16.56%)	17 (10.43%)	26 (15.95%)	6 (3.68%)	1.00 (0.00, 2.00)	3.00 (2.00, 5.00)	1.00 (1.00, 3.00)
	**p-value**	0.17	0.1	>0.99	0.65	0.45	>0.99	0.34	0.87	0.43	0.87	0.16	0.085	0.028	0.46
**Employment**	**N = 57**	16 (28.07%)	25 (43.86%)	28 (49.12%)	6 (10.53%)	9 (15.79%)	5 (8.77%)	3 (5.26%)	7 (12.28%)	1 (1.75%)	9 (15.79%)	0 (0.00%)	1.00 (0.00, 3.00)	4.00 (2.00, 5.00)	1.00 (0.00, 3.00)
	**p-value**	0.63	0.76	0.011	0.028	0.28	0.073	0.71	0.33	0.005	>0.99	0.045	0.65	0.27	0.72
**Food security**	**N = 55**	21 (38.18%)	34 (61.82%)	35 (63.64%)	12 (21.82%)	13 (23.64%)	10 (18.18%)	1 (1.82%)	11 (20.00%)	7 (12.73%)	17 (30.91%)	2 (3.64%)	1.00 (0.00, 3.00)	4.00 (3.00, 6.00)	2.00 (1.00, 3.00)
	**p-value**	0.25	0.01	<0.001	>0.99	0.72	0.84	0.47	0.55	0.81	0.002	0.74	0.11	<0.001	0.24
**Education**	**N = 53**	22 (41.51%)	26 (49.06%)	27 (50.94%)	9 (16.98%)	11 (20.75%)	4 (7.55%)	0 (0.00%)	9 (16.98%)	5 (9.43%)	11 (20.75%)	1 (1.89%)	1.00 (0.00, 3.00)	3.50 (2.00, 5.00)	1.00 (1.00, 3.00)
	**p-value**	0.07	0.65	0.006	0.46	>0.99	0.042	0.13	>0.99	0.64	0.31	0.31	0.87	0.15	0.89
**Transportation**	**N = 43**	16 (37.21%)	20 (46.51%)	18 (41.86%)	6 (13.95%)	7 (16.28%)	7 (16.28%)	1 (2.33%)	5 (11.63%)	2 (4.65%)	9 (20.93%)	2 (4.65%)	1.00 (0.00, 1.75)	4.00 (2.00, 5.00)	2.00 (0.75, 2.25)
	**p-value**	0.37	>0.99	0.29	0.23	0.42	>0.99	>0.99	0.38	0.19	0.37	>0.99	0.63	0.085	0.67
**Utilities**	**N = 41**	16 (39.02%)	16 (39.02%)	24 (58.54%)	7 (17.07%)	9 (21.95%)	6 (14.63%)	3 (7.32%)	8 (19.51%)	4 (9.76%)	11 (26.83%)	2 (4.88%)	1.00 (0.00, 2.75)	4.00 (3.00, 5.00)	2.00 (1.00, 3.00)
	**p-value**	0.27	0.39	<0.001	0.54	>0.99	0.82	0.38	0.65	0.8	0.064	>0.99	0.36	0.01	0.18
**Companionship**	**N = 39**	10 (25.64%)	23 (58.97%)	27 (69.23%)	7 (17.95%)	13 (33.33%)	5 (12.82%)	2 (5.13%)	10 (25.64%)	3 (7.69%)	11 (28.21%)	1 (2.56%)	1.00 (0.00, 3.50)	4.00 (3.00, 5.00)	1.00 (0.00, 3.00)
	**p-value**	0.46	0.12	<0.001	0.68	0.091	0.64	0.67	0.16	0.59	0.036	0.7	0.19	0.006	0.86
**Housing**	**N = 37**	13 (35.14%)	20 (54.05%)	23 (62.16%)	9 (24.32%)	7 (18.92%)	8 (21.62%)	1 (2.70%)	7 (18.92%)	4 (10.81%)	8 (21.62%)	2 (5.41%)	1.00 (0.00, 4.00)	5.00 (3.00, 6.00)	1.50 (0.00, 3.00)
	**p-value**	0.57	0.37	<0.001	0.67	0.83	0.48	>0.99	0.81	>0.99	0.34	>0.99	0.6	<0.001	0.69
**Health literacy**	**N = 31**	16 (51.61%)	17 (54.84%)	6 (19.35%)	11 (35.48%)	8 (25.81%)	11 (35.48%)	1 (3.23%)	7 (22.58%)	5 (16.13%)	3 (9.68%)	2 (6.45%)	1.00 (0.00, 2.00)	4.00 (3.00, 5.00)	1.00 (1.00, 4.00)
	**p-value**	0.012	0.34	0.072	0.061	0.64	0.009	>0.99	0.44	0.38	0.44	0.67	0.78	0.062	0.25
**Childcare assistance**	**N = 16**	2 (12.50%)	6 (37.50%)	6 (37.50%)	3 (18.75%)	2 (12.50%)	1 (6.25%)	0 (0.00%)	2 (12.50%)	1 (6.25%)	1 (6.25%)	1 (6.25%)	0.50 (0.00, 5.25)	2.50 (0.00, 4.00)	0.50 (0.00, 1.25)
	**p-value**	0.16	0.61	0.79	>0.99	0.54	0.32	>0.99	>0.99	0.7	0.48	0.59	0.78	0.044	0.041
**Legal**	**N = 15**	3 (20.00%)	7 (46.67%)	8 (53.33%)	0 (0.00%)	1 (6.67%)	2 (13.33%)	0 (0.00%)	1 (6.67%)	0 (0.00%)	5 (33.33%)	0 (0.00%)	0.00 (0.00, 1.00)	2.00 (1.00, 4.00)	1.00 (1.00, 2.00)
	**p-value**	0.41	>0.99	0.16	0.047	0.2	>0.99	>0.99	0.48	0.23	0.077	>0.99	0.26	0.18	0.59
**Received help**	**N = 166**	58 (34.94%)	82 (49.40%)	60 (36.14%)	38 (22.89%)	38 (22.89%)	30 (18.07%)	6 (3.61%)	31 (18.67%)	19 (11.45%)	30 (18.07%)	8 (4.82%)	1.00 (0.00, 2.00)	3.00 (2.00, 5.00)	1.00 (0.00, 3.00)
	**p-value**	0.1	0.2	0.42	0.54	0.76	0.61	0.54	0.4	0.85	0.39	0.78	0.56	0.006	0.88

Transportation insecurity was significantly associated with English-speaking patients (67.44% vs 48.42%, p = 0.030). The need for childcare assistance was significantly more likely to be reported by younger patients (mean age: 32.0 vs 39.5, p = 0.022), patients taking fewer mean medications (0.5 vs 1.0, p = 0.041), and patients with a lower mean number of comorbidities (2.5 vs 3.0, p = 0.044). Employment instability was significantly associated with alcohol use (47.37% vs 28.99%, p = 0.011) and psychiatric illness (49.12% vs 29.95%, p = 0.011). This was also significantly associated with patients who did not have Diabetes Mellitus (DM) (24.64% vs 10.53%, p = 0.028), or who did not take a DM (14.49% vs 1.75%, p = 0.005) or dyslipidemia medication (6.76% vs 0%, p = 0.045). Unmet education needs were significantly more likely to be reported by younger patients (30 vs 40, p = 0.012), patients with psychiatric illness (50.94% vs 29.86%, p = 0.006), and patients without dyslipidemia (19.43% vs 7.55%, p = 0.042). Patients without DM were significantly more likely to request legal resources (22.89% vs 0%, p = 0.047).

Unmet companionship needs were significantly associated with psychiatric illness (69.23% vs 28.00%, p<0.001), taking a psychiatric medication (28.21% vs 14.22%, p = 0.036), and a higher mean number of comorbidities (4 vs 3, p = 0.006). Health literacy needs were significantly associated with dyslipidemia (35.48% vs 14.59%, p = 0.009), gastrointestinal disease (51.61% vs 28.33%, p = 0.012), female (83.87% vs 64.81%, p = 0.041), Hispanic/Latino (54.84% vs 30.47%, p = 0.014), Spanish-speaking (45.16% vs 24.89%, p = 0.030), and older patients (mean age: 47 vs 38, p = 0.012). Patients with a higher mean number of comorbidities (3.0 vs 2.5, p = 0.006) were significantly associated with requesting a community referral to address an unmet social need of any kind.

Univariate analyses were also performed to identify significant associations between lab values (Table 6) with social needs or receiving resources. No significant associations were found for patients who reported unmet employment, education, companionship, childcare assistance, or legal needs. There were significantly lower mean high-density lipoprotein (HDL) cholesterol levels in those who reported food insecurity compared to those who did not (31 vs 48, p = 0.006). There were significantly lower mean hemoglobin A1c levels in those who reported unmet financial needs compared to those who did not (5.9 vs 6.3, p = 0.039). There was also significantly lower mean systolic blood pressure (SBP) in those who reported unmet transportation needs compared to those who did not (119 vs 128, p = 0.033). Finally, there were significantly higher mean total cholesterol levels in those who reported health literacy needs compared to those who did not (224 vs 197, p = 0.037).

**Table 6:** Objective measurements of the patients who filled out the socioeconomic screening questionnaire.

Patient Measurements	Mean Value
**Vitals**	
Systolic Blood Pressure, mm Hg	127.21 ± 1.39 (118.00, 135.00)
Diastolic Blood Pressure, mm Hg	80.41 ± 0.98 (73.50, 86.00)
Heart Rate	80.00 ± 1.10 (71.00, 89.00)
Body Mass Index	28.91 ± 0.72 (23.25, 34.44)
**Labs**	
Triglyceride	220.63 ± 28.15 (125.00, 277.00)
Total Cholesterol	207.30 ± 7.43 (189.00, 228.00)
Low-Density Lipoprotein	148.61 ± 15.98 (108.00, 151.00)
Very Low-Density Lipoprotein	71.30 ± 20.51 (25.00, 44.00)
High-Density Lipoprotein	46.20 ± 2.08 (42.00, 55.00)
Hemoglobin A1c	6.80 ± 0.26 (5.80, 6.92)
Glomerular Filtration Rate	87.35 ± 2.50 (81.00, 97.00)
Blood Urea Nitrogen	13.94 ± 0.78 (11.00, 18.25)
Creatinine	0.84 ± 0.03 (0.71, 0.98)

## Discussion

We present an innovative social intervention model in which medical students were mobilized to assess SDH factors afflicting underserved patients in several free community health clinics. Using a validated socioeconomic screening questionnaire, we identified the unmet needs of patients seen in our SRFCs and found that 63% of patients requested assistance finding community resources. Overall, the most reported SDH factor was unmet financial needs (62%) which were significantly associated with a higher number of comorbidities. Our results support utilizing poverty as an efficient, highly sensitive screening topic to identify at-risk patients in need of social resources [17].

Individual-targeted assessments of detrimental SDH factors are effective and promising methods of mitigating key drivers of health inequities [8]. A 2019 systematic review and meta-analysis demonstrated that the health behavior benefits of social interventions were significantly related to improvements in overall well-being, hemoglobin A1c levels, and cessation of alcohol/tobacco misuse (p<0.05) [18]. Absence of these interventions has also been identified as a critical contributor in primary nonadherence to chronic disease medication [19]. However, screening by primary care providers is often limited by their short appointment times, disproportionately high workloads, and management of related administrative responsibilities [13]. Mobilizing medical students as social workers lessens these burdens through the efficient allocation of bureaucratic duties within busy clinics [8, 13, 20]. This included directly assisting patients with complex insurance application forms and integrating community resources to empower future self-sufficiency outside of the clinic [4, 13].

Patients with Type 2 DM who are connected to community social resources report improved self-care, quality of life, and improved glycemic control [1]. Gorrindo et al. demonstrated that appointing even a preclinical first-year medical student to educate SRFC patients about their health and other non-medical needs resulted in significantly improved hemoglobin A1c levels, (9.6 to 7.9, p<0.0001) [21]. Similar to our model, Dadlani et al. also implemented a system to screen for detrimental SDH factors at SRFCs. Their most requested resources were reported as utilities (18%) and mental health (18%), which was slightly higher than our clinics (16% and 15%, respectively) [22]. In contrast, our study identified financial resources as the most common unmet need (62%) and focused on associations between social needs and medical conditions, whereas Dadlani et al. utilized patient zip codes to identify geographic gaps in the distribution of resources [22].

Our results indicate a significant association between reporting unmet social needs and the presence of more cardiovascular risk factors. Notable associations included childcare assistance with the number of comorbidities or medications, food security with the number of comorbidities, housing instability with tobacco use, transportation with SBP, and financial needs with hemoglobin A1c levels. One likely possibility is that patients who are chronically sick and financially disadvantaged are exhausted from the overwhelming wait times in the current healthcare system [23]. Progressively worsening chronic health conditions result in additional appointments that require more time and resources than underserved patients are equipped to meet, leading to avoidance of seeking social services [8, 23]. The significant association between employment stability and DM, taking a DM medication, or taking a dyslipidemia medication may further imply that these patients are forced to work in order to afford the financial burden associated with their chronic diseases [24].

However, there were predictably higher significant mean total cholesterol and lower mean HDL levels seen in patients who reported unmet health literacy and food security needs, respectively. These exceptions could be due to the immediate necessity of food or difficulty interpreting the screening tool at the appointment [3, 8]. We demonstrated significantly more requests for community resources after a simple conversation during a time when patients would be waiting regardless. Given the disparity between cardiovascular disease and the active medication list of our cohort (especially antiplatelet therapy), our results strongly suggest that medical student-facilitated social intervention may contribute to lowering the morbidity and mortality rates associated with cardiovascular disease [25]. A higher number of comorbidities and a history of psychiatric illness were the two most common medical problems significantly associated with several unmet social needs and may be an area of interest for future models.

It is well documented that patients with more chronic health conditions suffer from a higher socioeconomic burden [1]. Psychosocial factors similar to those in our study are responsible for exacerbating the baseline risk factors that cause cardiac events and long-term disability [26]. Neglected complications of chronic, untreated peripheral vascular involvement can also lead to life-altering foot ulcers, especially in minority and socially disadvantaged patient populations [27]. The resulting impact on ambulation and finances can be emotionally overwhelming, and even devastating to patients who inevitably require amputations [24]. Strategies aimed at preventing these outcomes in future, larger social interventions should reflect the needs of the targeted community through a more expansive assessment of well-known risk factors such as tobacco use, family medical history, and metabolic syndrome [26]. Preemptive identification and appropriate management of physical, mental, and social care is therefore critical to improving disproportionate disparities in long-term health outcomes [1, 26, 27].

### Strengths

Strengths of this study include an innovative, systematic approach to address often overlooked SDH factors in a vulnerable population. Our results also provide evidence-based guidance for others seeking to do the same. This further benefits the education of developing physicians by emphasizing the importance of delivering holistic healthcare through community outreach. Similar community service-based opportunities further benefit medical students by reinforcing cultural competence, team-based learning, and responsibility to advance health equity [10].

### Limitations

Generalization of the results are limited given the small sample size and retrospective review limited to a single community center. The collection of some charted information was also dependent on the unstandardized and widely varying documentation performed by medical students. Finally, the patient’s pre-appointment resource status may be a confounding variable as we included telehealth and in-person visits during the COVID-19 pandemic, which may have selected for patients who were able or willing to seek treatment during that time.

## Conclusions

In summary, we identified a high prevalence of synergistic cardiovascular and psychiatric comorbidities in underserved patients that was complicated by several underlying unmet social needs. We found that 63% of participants requested assistance finding community resources. While the most reported SDH factor was unmet financial needs (62%), a higher number of comorbidities and history of psychiatric illness were the two most common medical problems significantly associated with several unmet social needs. Future research can focus on larger-scale, prospective patient health outcomes based on the success of community referrals aimed at addressing the unmet psychosocial needs of underserved patients.

## Supporting information

S1 Data Minimal data set is available as supporting information file. (XLSX)
