# Correction: Assessing social needs among patients with cardiovascular and psychiatric comorbidities in free community health clinics

**DOI:** 10.1371/journal.pone.0301576

**Published:** 2024-03-28

**Authors:** David Haddad, Venkata Sai Jasty, Jacob Ref, Paul Hsu, Patricia Lebensohn, Tze-Woei Tan

## Notice of Republication

An incorrect version of S1 Data File was published in error. This article was republished on March 19, 2024, to correct this error. Please download this article again to view the correct version.
